# Introducing Tempeh as a New Plant-Based Protein Food Item on the Danish Market

**DOI:** 10.3390/foods10112865

**Published:** 2021-11-19

**Authors:** Margit Dall Aaslyng, Rikke Højer

**Affiliations:** Nutrtion and Health, University College Absalon, Sdr. Stationsvej 30, 4200 Slagelse, Denmark; rho@pha.dk

**Keywords:** tempeh, reducing meat intake, consumer behaviour, home use test, plant protein, RATA, fermentation

## Abstract

Decreasing meat consumption has resulted in a need for new high-quality protein sources. Tempeh is relatively unknown in Denmark and might be capable of meeting this need. The aim of the study was to describe the success criteria for introducing locally produced tempeh and to investigate the sensory quality of three types of tempeh. Only 24% of the consumers in the survey (*n* = 395) used meat alternatives, which might be explained by a low level of satisfaction with availability. Tempeh was known by 26%—the less meat eaten, the greater the knowledge of tempeh. Twenty-three per cent of the consumers had positive attitudes towards tempeh. The three types of tempeh had markedly different sensory profiles. Nevertheless, the home use test showed that they could be used interchangeably in different recipes. In addition, the consumers were more positive about recipes in which tempeh did not resemble meat compared with meat-inspired recipes. In conclusion, introducing locally produced tempeh on the Danish market is possible but would require further knowledge of the product. In addition, tempeh should be sold as a tasty, high-quality protein food item in its own right. Recipes using tempeh should reflect this and not mimic meat recipes.

## 1. Introduction

A considerable part of the population either want to minimise their meat intake by being vegan or vegetarian or simply reduce their meat intake by being flexitarian or meat reducers [1]. This has resulted in a need for high-quality protein sources that also contain other nutritious compounds such as vitamins and minerals, especially vitamin B12 [2].

Tempeh is a traditional Indonesian food item, which, in its original version, is dehulled acid-soaked soy beans solid state fermented with the mold Rhizopus spp. This results in a white cake-like structure which can be sliced and prepared in various ways, e.g., fried or baked. Even though tempeh is originally made from soy beans, other sources are used as well: other bean types [3,4], lentils [3], rapeseed press cake [5], lupin [6], maize [7] and barley [8], resulting in a variation in both nutritional properties and sensory quality [3].

The fermentation increases the nutritional quality of the protein source by reducing the content of antinutritional components [9], increasing the content of the B-vitamin group including B12 [6,10], increasing the protein digestibility measured by in vitro protein digestibility [7] and improving the amino acid composition [7,11]. Furthermore, health-promoting compounds such as γ-Aminobutyric acid [12] are formed during fermentation.

In addition to the nutritional improvement, the fermentation changes the sensory properties significantly, resulting in a very different product that is suitable for other applications than the unfermented beans. Even though tempeh is a food item, few studies have included sensory testing, Vital et al. [4] being one of them. Instead, the focus is mainly on the nutritional value (e.g., [9,12]). However, sensory quality is a crucial factor when introducing new food items on the market, and research into the sensory quality of tempeh is therefore necessary.

Tempeh is not widespread in Europe, and although relatively well known in the Netherlands, it is almost unknown in Denmark. Since consumers in Denmark have the same trend going towards reducing their meat consumption, not unlike the rest of the world [13], tempeh could be an attractive protein source to introduce to the Danish consumers. However, introducing new food types as substitutes for meat is not a straightforward process. Tucker [14] identified price and sensory quality as the main obstacles to eating less meat. In addition, she stresses that lack of knowledge on how to cook dishes without meat was also an important factor. Hoek et al. [15] showed that consumers who do not eat meat substitutes preferred new products that resemble meat, while heavy users of meat substitute products had positive attitudes towards products that differed from meat in their sensory properties. However, the sensory quality of the products was important for all groups of consumers. When aiming to introduce new food items as substitutes for meat, it is important to focus on the sensory quality, price and recipes in which the product both resembles meat and is, in itself, unique.

The aim of this study was to investigate the optimal way of introducing locally produced tempeh to the Danish market. Initially, the main success criteria were described on the basis of an internet-based survey identifying consumer product knowledge, attitudes toward tempeh as a meat substitute and the potential for introducing tempeh. This was followed by a sensory description of three types of locally produced tempeh and two home use tests. First, one variation of tempeh was tested in three different recipes, and then the three different varieties of tempeh were tested in two recipes. This was completed in order to evaluate whether the recipe affected attitudes towards tempeh, and to distinguish between the potentials of the three different types of tempeh.

## 2. Materials and Methods

The tempeh used in all of the experiments was produced by the company Contempehrary. Contempehrary (www.contempehrary.com (accessed on 1 November 2021)) is a small Danish company that produces tempeh using Nordic ingredients, and is one of only two companies that produce tempeh in Denmark. They produce three different types of tempeh: one made with yellow split peas with a protein content of 12% (pea); one made with fava beans with a protein content of 13% (fava beans); and the third kind made with a combination of barley, oats, rye and hemp with a protein content of only 6% (barley). All three kinds of tempeh are produced by a lactic acid fermentation followed by a solid state fermentation using Rhizopus spp.

All data analysis was performed using R and RStudio version 1.2.5033 and the packages lm4, lmer and candisc.

### 2.1. Survey

An internet-based questionnaire was made in SurveyXact [16], and a link was shared using SoMe (Facebook and LinkedIn). A total of 395 respondents answered both the consent question and the questions related directly to tempeh, while 348 answered all questions. The respondents were distributed across all regions of Denmark.

The questionnaire was divided into eight demographic questions (consent, gender, age, region, education, civil status, children, children at home), two food habit questions (self-reported eating habits, shopping habits) and up to 13 questions about tempeh and plant-based meat substitutes (the number depended on the answers) answered on a 5-point categorical scale: ‘not at all’, ‘to a small extent’, ‘neither/nor’, ‘to some extent’, ‘to a great extent’ and—for questions related to the use of tempeh—‘don’t know’. Finally, one question concerned the impact of 17 different factors on the choice of plant-based meat analogues, rated on a scale from 0 to 10. The full questionnaire can be seen in the Supplementary Materials section.

The data were analysed using a descriptive analysis combined with an analysis of variance with eating habit (omnivore or not) as a fixed effect when appropriate.

### 2.2. Sensory Description Using Rate-All-That-Apply

Untrained assessors (*n* = 44), all students at University College Absalon, evaluated the sensory properties of the three types of tempeh using rate-all-that-apply (RATA). The attributes were defined on an evaluation performed by a panel of experts, all of whom were familiar with the products and with sensory assessments. The appearance attributes were: fried, yellow, green, brown, dry and crispy. The flavour and taste attributes were: bitter, nut, sweet, mushroom, sour/acidic, fermented, rancid, earth, rye bread, smoke and astringent. The texture attributes were: dry, floury, sticky and crumbly. In addition, they assessed the more general aftertaste attributes: intensity and long-lived. The order of the attributes was following the eating experience (first appearance, then taste/flavour and, finally, texture) instead of being in a balanced order as often proposed [17,18]. The assessors initially checked whether or not an attribute described the actual tempeh sample. If it did, they were asked to indicate the intensity on a scale from one (little) to three (much). If an attribute was not checked, its value was set at zero. 

The tempeh was cut into slices measuring 1 × 2.5 × 4 cm, smeared with grapeseed oil and either baked in an oven at 175 °C for 7 min or fried on a hot pan for 1.5 min. All three samples from the same cooking method were served together, and therefore two servings were given. The order in which the three tempeh samples were assessed differed between assessors and followed a block design. Half of the assessors evaluated the baked samples first, while the other half evaluated the fried samples first.

A mixed model analysis of variance was performed on all attributes, with cooking and tempeh type, and the interaction between these, as fixed effects, and assessor as random effect followed by a canonical variate analysis.

### 2.3. Home Use Test

The freshly produced tempeh was frozen for a maximum of two days before use. In the first HUT, tempeh made with pea was used. In the second HUT, all three types of tempeh were used. The full instruction as well as the questionnaire is given in the Supplementary Materials section.

#### 2.3.1. HUT 1: The Effect of Recipe on Attitudes towards Tempeh

Young consumers aged between 20 and 29 years were recruited using SoMe. They all lived in Copenhagen or in surrounding areas. When recruited, the consumers were asked to choose between three recipes (Table 1): Nordic inspired summer rolls, fried tempeh or tempeh ‘meatballs’. Furthermore, they could indicate the number of people in their household (between 1 and 4). A total of 107 households and 159 consumers participated.

On the test day, a bag containing all the ingredients for the chosen recipe was delivered to their household. After cooking and evaluating, they had to report back using SurveyXact (Rambøll, Aarhus, Denmark, 2019) on a 9-point hedonic scale for liking, rating generally and for specific attributes, and also for how easy the cooking was. The full questionnaire can be seen in the Supplementary Materials section. The data were analysed using a mixed model with dish as a fixed effect and consumer as random effect. 

#### 2.3.2. HUT 2: Potential of the Three Types of Tempeh Used in Two Recipes

Young consumers aged between 20 and 29 years were recruited using SoMe. They all lived in Roskilde, a suburb of Copenhagen, and the surroundings. When recruited, they could choose between a tempeh pizza and tempeh in filo dough rolls (Table 1). At a specific time slot, the consumers could pick up a bag containing all the ingredients from a central location. A total of 116 consumers from 80 households participated.

All of the consumers evaluated all three types of tempeh in three sections of the pizza or three different filo dough rolls. After cooking and evaluating, they had to report back using SurveyXact [16] on a 9-point hedonic scale for liking, rating generally and for specific attributes, and also for how easy the cooking was. The full questionnaire can be seen in the Supplementary Materials section. The data were analysed using a mixed model with dish as a fixed effect and consumer as random effect.

## 3. Results

### 3.1. Survey

The respondents of the questionnaire were mainly young people, with 58% aged between 18 and 29 years, and only 4.5% older than 60 years. Most of the respondents (78%) were female, 22% were male, and one respondent did not wish to classify their gender. Eighty percent had a high school education, a bachelor’s degree, a master’s degree or a PhD as their most recently completed education. This means that most of the respondents were young, well-educated people. Two thirds (67%) lived together with a partner, and 26% had children living with them.

When describing their eating habits, they could describe themselves as omnivore (67%), flexitarian (20%), lacto-ovo-vegetarian (3%), ovo-vegetarian (1%), pescatarian (5%) or vegan (4%). Due to the low number of respondents in the different meat-free categories, these were merged in the data analysis. 

Knowledge of tempeh was relatively low (Table 2), although it was expected that those choosing to answer the questionnaire would have more knowledge of tempeh than the population in general. A clear trend was seen: the less meat and other animal products you eat, the greater your knowledge of tempeh. Of those who knew about tempeh, 19% had eaten it at least seven times, 26% had eaten it between three and six times while 22% has tasted it one or two times. The rest (33%) had never tasted tempeh even though they knew about it. This means that even though 26% of the respondents knew about tempeh, most of the respondents had never tasted tempeh or had only tasted it a few times.

Of the consumers who had tasted tempeh, 56% wanted to use tempeh for cooking either to some extent or to a great extent, 58% thought it was a suitable protein source, 68% thought that it was a sustainable food item, and 65% stated that they could eat it for climate reasons. However, within this group, the vegetarians/vegans found it more sustainable and would use it to a greater extent than the flexitarians and omnivores (*p* = 0.02) and were especially more positive about eating it for climate reasons (*p* < 0.001).

All participants were asked if they were satisfied with the availability of meat alternatives in general, if they used them and how much they liked them (Table 3). Even though half of the respondents answered that they liked the plant-based meat alternatives, only 24% answered that they used them. This might be explained by the fact that only 33% answered that they were satisfied with the availability. Again, the answers also depended on the respondents’ eating habits, with vegetarians/vegans being more positive and satisfied and using them more often than flexitarians and omnivores (*p* < 0.001).

**Figure 1:**
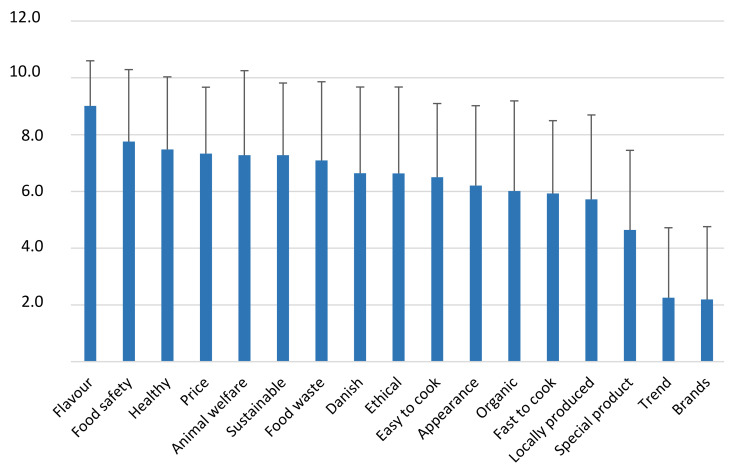
Importance of drivers for choice of plant-based meat alternatives rated on a scale from 0 (not important) to 10 (very important). Average and standard deviation are given.

To identify the drivers of choice for plant-based meat alternatives, the respondents were asked to rate different factors related to intrinsic and extrinsic factors (Figure 1). Taste and flavour (in Danish this is the same word) were rated as the most important factor, indicating that the sensory quality is crucial. Food safety, health and price are also important factors related directly to the respondents, while sustainability and animal welfare are important external factors. In contrast, trends and brands scored low and were considered unimportant for the respondents.

### 3.2. Sensory Description Using Rate-All-That-Apply

No significant interaction was seen between tempeh type and cooking method for any attributes (*p* > 0.05). Frying was more intensive than oven baking for three attributes: fried appearance (fried: 1.6, oven: 0.6, *p* < 0.001), smoke flavor (fried: 0.5, oven: 0.3, *p* = 0.03) and crispy (fried: 0.9, oven: 0.5, *p* < 0.001). No difference was seen between cooking methods for the rest of the attributes. 

**Figure 2:**
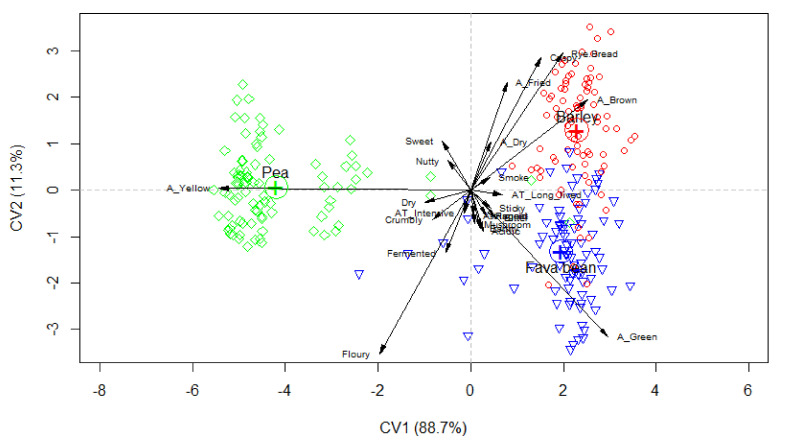
Different sensory descriptions of three types of tempeh (Fava bean: blue triangle; Pea: green rhombus; and Barley: red circle) assessed by rate-all-that-apply and analysed using canonical variate analysis.

The three different types of tempeh had very different sensory profiles, and they were clearly differentiated on the plot (Figure 2). The main difference was in appearance, pea being yellow, fava bean being green and barley being brown, fried and with a crispy surface. The differences in flavour were fewer and smaller. The main difference was that the barley tempeh had a distinct flavour of rye bread. Furthermore, the barley and pea tempeh were slightly sweeter and nuttier than the fava bean tempeh, while the fava bean and pea tempeh were more fermented than the barley tempeh. The main texture attribute was floury, which was used to describe the fava bean and the pea tempeh to a much greater extent than the barley tempeh.

### 3.3. Home Use Test

#### 3.3.1. The Effect of Recipe on Attitudes towards Tempeh

For the evaluation of the effect of recipe, tempeh based on peas was used as this tempeh type was regarded as the most neutral, sweet and nutty variant of the three types of tempeh (Figure 2). The participants had the possibility of choosing which recipe they wanted to try. Only 23% of the participants knew about tempeh beforehand, and only 8% had tried to use it before. Prior knowledge was, therefore, sparse.

The Nordic inspired summer rolls were the most popular dish chosen by the majority of the households, with 71 out of 107 households giving 108 consumer evaluations. On the other hand, tempeh ‘meatballs’ were only chosen by ten households, resulting in 12 evaluations (Table 4).

The summer rolls were generally given the most positive evaluation. The consumers liked the dish and the tempeh in the dish significantly more than the other two dishes (Table 4). In addition, it better met their expectations, and the balance and richness in flavour and taste were better. In comparison, no significant difference was seen between fried tempeh and tempeh ‘meatballs’. In these results, it must be taken into account that only 12 consumers evaluated the ‘meatballs’, which is a rather small group of consumers to take any conclusion from.

#### 3.3.2. Potential of the Three Types of Tempeh in Two Recipes

In order to evaluate if there was an interaction between the different types of tempeh in different recipes (i.e., one type would be better suited to a certain type of recipe than one of the other types), a new HUT was performed, providing the consumers with a choice between two recipes but assessing all three types of tempeh in the one they chose. The questions used were the same as those in the first HUT.

More consumers chose the recipe with filo dough than the pizza (Table 5). No significant interaction between dish and tempeh type was found except for balance in flavour and taste (*p* = 0.01). Here, a clear significant difference between the tempeh types was seen for pizza, while no difference was seen for filo dough. This means that, for most of the attributes, the evaluation of the tempeh types was independent of the dish. The analysis was therefore repeated for each dish individually. 

Only few significant differences were seen between the three tempeh types. The umami taste was rated lower in the pea tempeh than in the other tempeh types, and a tendency (*p* = 0.06) was seen for pizza with barley to be liked more than the other pizza types. However, differences between dishes and between tempeh in the dishes were generally rather small. The average assessment of the attributes was neutral—between 4 and 6 in assessment—with only appetising appearance and flavour/taste being positive (above 6). Across the two types of dishes and the three types of tempeh, 23% were positive (answered 7, 8 or 9), 31% neutral (answered 4, 5 or 6), while 47% were negative (answered 1, 2 or 3) towards using tempeh in another dish. This shows that, independently of the average neutral answers, a clear segment of 23% were positive towards using tempeh in the future and can thus be considered a potential tempeh user segment.

**Figure 3:**
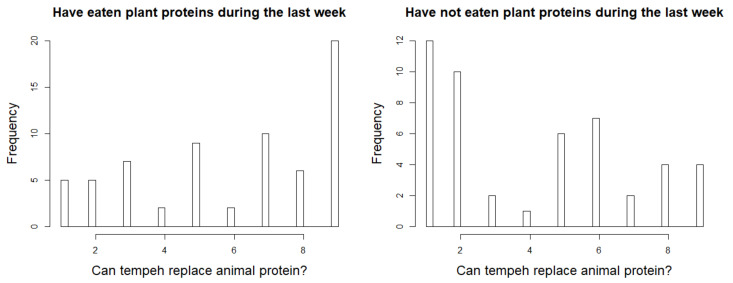
Histogram showing the intention of using tempeh in another dish depending on whether or not consumers have eaten plant proteins during the past week, assessed on a scale from ‘not suitable’ (1) to ‘very suitable’ (9).

Additionally, the consumers were asked to what extent they thought tempeh could replace animal protein. As seen in Figure 3, the answer clearly depended on whether or not they had eaten plant proteins for dinner during the past week. Those who had eaten plant protein during the past week were clearly positive about substituting animal protein with tempeh, while the opposite was the case for those who had not eaten plant protein for dinner.

## 4. Discussion

The Danish population are relatively unfamiliar with tempeh, as confirmed by the survey which revealed that only 26% knew about tempeh. The less meat you eat, the greater the probability that you will know about tempeh. Tucker [14] describes that, while health concerns are one of the reasons people reduce their meat intake, health is also mentioned as a concern when people reduce their meat intake. This might explain why more vegetarians and flexitarians know about tempeh than omnivores, since the meat-free or meat-reduced diet groups could have an active approach to finding other high protein quality food sources. With more and more people switching to meat-free or meat-reduced diets [1], this trend will therefore naturally result in an increased demand for tempeh. This is further strengthened by the fact that 33% of the respondents in the survey were not satisfied with the current availability of meat analogues on the market. Furthermore, those consumers who had eaten plant proteins during the past week were positive towards tempeh as a substitute for meat protein, while those consumers who had not eaten plant proteins during the past week were negative (Figure 3).

The following drivers for eating plant-based protein sources instead of meat have been identified: eating quality, price and food safety as internal factors, and animal welfare and sustainability as external factors (Figure 1). These must be taken into account when introducing tempeh. However, it is also of interest that Tucker [14] describes unfamiliarity with cooking of meat analogues and concerns about nutrition as the main barriers to consumers using more plant-based meat analogues. Therefore, it can be concluded that a good flavour/eating quality, an acceptable price and recipes preferably containing information on nutrition and sustainability can be seen as a basis for introducing tempeh on the Danish market.

The main target group in the home use test was young people, which corresponds to Malek and Umberger (2021), who show that a greater proportion of meat reducers, vegetarians and vegans were aged under 34 years. Even though tempeh is for all consumers and not just meat-free diet consumers, the more positive attitude identified in the survey from the latter group shows that they could be the front-runners in integrating tempeh as a new food item in their daily diet. The dishes were constructed based on the concept of ‘flavour principles’ presented by Rozin and Rozin [19], which suggests that combining a familiar flavour with an unfamiliar new food may increase a willingness to try it. Furthermore, the dishes varied in flavour familiarity, ranging from high flavour familiarity (meat balls with pasta dish) to low flavour familiarity (miso marinade dish) to investigate consumer preferences. Evaluation of the dishes were based on the concept culinary success factors for determining the palatability of food as defined by Klosse et al. [20]: (1) expectation and palatability, (2) smell and palatability, (3) balance of gustatory flavours and palatability, (4) umami and palatability, (5) mouthfeel and palatability, and (6) flavour richness and palatability.

The target group did not see tempeh as a direct substitute for meat choosing the most familiar dish (as in tempeh ‘meatballs’), but rather as a food item in its own right as in summer rolls, which was the most popular recipe and also the one given the most positive assessment. This is supported by Vital et al. [4], who found low buying intention for a tempeh burger, since it was too far removed from an ordinary burger and yet still resembled it, and Hoek et al. [15], who showed that users of meat substitutes wanted products that were different from meat in their sensory properties, while consumers who do not eat meat substitutes today preferred new products that resemble meat. When developing recipes for meat reducers/meat avoiders, the focus should therefore be on tempeh as a food item in its own right and not as a direct substitute for meat.

In the second HUT, consumers could also choose between a well-known recipe (pizza) and a less familiar recipe (tempeh in filo dough), and, as in the first HUT, most people chose the less familiar recipe, supporting the fact that this target group preferred recipes that did not resemble meat. Even though the three types of tempeh were clearly different in their sensory properties (Figure 2), only minor differences were seen in the liking of the dish and the liking of the tempeh in the HUT (Table 5). In the development of recipes for tempeh, general recipes can be developed without taking into account the types of tempeh they use. 

Preference for novel food can be seen as a combination of opposing motives divided into approach and avoidance [21]. In the case of tempeh, one clear approach will be a suitable, tasty, high-quality protein food item for meat reducers/meat avoiders. Since this target group have already chosen to reduce their consumption of meat for reasons of animal welfare and/or sustainability, tempeh as a locally produced plant-based protein source meets their external needs. In terms of the average liking of tempeh, a medium to slightly negative assessment was seen, especially in the last HUT, and the sensory properties might be part of the avoidance behaviour. However, 23% of the consumers were clearly positive and therefore represent a clear segment for introducing tempeh in Denmark.

Tempeh can be seen as a new and nutritious food item on the Danish market. The tempeh used in this study was produced from local protein sources instead of soy beans, and resulted in a protein content of 12–13% for the pulses-types and 6% for the barley type. This is lower than what can be expected in traditional tempeh made from soy beans which is between 20% [22] and 25% [3]. However, the fat content of the tempeh in this study is also markedly lower, being up to 3% while it is reported to be 11% [3] in the soy bean based tempeh. Future studies should further evaluate the nutritious value of Danish tempeh based on local ingredients.

## 5. Conclusions

Locally produced tempeh in Denmark can be introduced on the Danish market by targeting meat reducers/meat avoiders, who today are primarily young consumers. Of these consumers, 23% were very positive towards tempeh and can be seen as the main target group. Since tempeh is relatively unknown among Danes today, it is important to increase consumers’ knowledge of tempeh and its uses. However, for this consumer group, tempeh should not be sold as a meat substitute, but rather as a tasty, high-quality protein-rich food item in its own right. Any recipes that are developed should therefore reflect this and not mimic meat recipes.

## Figures and Tables

**Table 1:** Recipes for the home use tests 1 and 2.

	Full Name of Dish	Recipe (1 Person)	
HUT 1			
Nordic inspired summer rolls	Nordic summer rolls with teriyaki marinated tempeh and sweet and sour and peanut dip	Marinade:	Peanut butter dip:
		0.5 dl Teriyaki	0.5 dl Crunchy peanut butter
		½ clove of garlic	0.25 dl water
		¼ chopped chili	½ a lime
		0.25 tbsp grated ginger	1 tbsp soy sauce
			salt and pepper
		Stuffing:	
		100 g tempeh	Sweet and sour dip:
		4 pieces rice paper	0.5 dl water
		75 g carrots	2 tbsp sugar
		2 asparagus spears	2 tsp vinegar
		1 spring onion	¼ chili
		1/3 of a cucumber	2 tbsp fish sauce
		50 g red cabbage	Juice of ½ a lime
		30 g radishes	
		¼ bunch cilantro	
		1 lime	
		Oil for frying	
Marinated fried tempeh	Fried tempeh with green asparagus, orange-miso sauce, black sesame and cauliflower rice	100 g tempeh	Cauliflower rice:
		6 green asparagus spears	175 g cauliflower
		Oil for frying	The bottom of the green asparagus
			Salt
		Miso sauce:	
		1 tbsp miso paste	Decorate/sprinkle:
		2 tbsp balsamic vinegar	1 tbsp black sesame seeds
		2 tbsp oil	
		Juice of ½ an orange	
Tempeh ‘meatballs’	Tempeh ’meatballs’ with parsley and hazelnuts served in tomato sauce with pasta	Tempeh ‘meatballs’:	Tomato sauce:
		100 g tempeh	1 can of whole peeled tomatoes
		¼ bunch of parsley (only leaves)	¾ onion
		¼ clove of garlic	1¾ clove of garlic
		¼ onion	3 parsley stalks
		10 g hazelnuts	½ tbsp oil
		2 tbsp water	½ tsp salt
		A pinch of salt	
		½ tbsp oil	Pasta:
			100 g whole grain pasta
			1 tsp salt for boiling water
**HUT 2**			
Tempeh pizza	Pizza with tempeh, potato, Jerusalem artichokes and pickled red onions topped with crispy marinated kale
		Pizza dough:	Pickled red onions:
		150 g wheat flour tipo 00	150 g red onion
		5 g yeast	1 tbsp oil
		1 dl water	40 g sugar
		1 tbsp olive oil	0.5 dl vinegar
		½ tsp salt	Salt, pepper
		Pizza filling:	Crispy marinated kale:
		30 g (3 slices) pea tempeh	40 g kale
		30 g (3 slices) bean tempeh	1 tbsp olive oil
		30 g (3 slices) grain tempeh	½ tbsp vinegar
		2 tbsp olive oil	½ tsp sugar
		220 g potatoes	¼ tsp mustard
		125 g Jerusalem artichokes	Salt, pepper
		1 clove of garlic	
		4 parsley stalks	
		0.5 dl olive oil	
Tempeh in filo dough	Baked tempeh in crispy filo dough with pumpkin, mushrooms and potato. Sauce vert on the side	Filo dough:	Stuffing (cont.):
		3 sheets of filo dough	75 g mushrooms
		3 tbsp olive oil	1 tbsp oil
			2 parsley stalks
		Tempeh:	Salt, pepper
		30 g (2 slices) pea tempeh	
		30 g (2 slices) bean tempeh	Sauce vert:
		30 g (2 slices) grain tempeh	1 dl vegan yoghurt
		1 tbsp olive oil	1 tbsp olive oil
			½ tsp sugar
		Stuffing:	4 stems of mint
		50 g red onion	2 parsley stalks
		150 g pumpkin	Salt, pepper
		150 g potatoes	

**Table 2:** Knowledge of tempeh depending on eating habits (% of all rows).

	Do You Know About Tempeh?	
	Yes	No
Omnivore	17%	83%
Flexitarian	31%	79%
Vegetarian/vegan	68%	32%
All	26%	74%

**Table 3:** Satisfaction with the availability of plant-based meat alternatives, if they use plant-based meat alternatives, and how much they like plant-based meat alternatives (% of participants).

Plant-Based Meat Alternatives:	Not at All or to a Small Extent	Neither/Nor	To Some or a Great Extent
All			
Availability	29	38	33
Use	70	6	24
Liking	29	20	51
Omnivore			
Availability	42	33	25
Use	83	5	12
Liking	34	23	43
Flexitarian			
Availability	23	36	40
Use	51	11	38
Liking	21	18	62
Vegetarian/Vegan			
Availability	19	15	67
Use	27	8	65
Liking	17	10	73

**Table 4:** Assessment of a dish with tempeh rated on a scale from 1 (not at all) to 9 (very much). The number of consumers is given under each dish. The average is given +/− stderr. Different letters in the same line show a significant difference.

Attributes	Summer Rolls (*n* = 108)	Fried Tempeh (*n* = 39)	Tempeh ‘Meatballs’ (*n* = 12)	*p* (Difference)
Liking of the dish	7.1 ^a^ (0.2)	5.6 ^b^ (0.3)	5.6 ^b^ (0.5)	*p* < 0.001
Liking of tempeh in the dish	6.2 ^a^ (0.2)	5.1 ^b^ (0.4)	4.8 ^b^ (0.7)	*p* = 0.01
Tempeh was suitable in the dish	6.7 ^a^ (0.2)	6.3 ^a,b^ (0.4)	5.0 ^b^ (0.6)	*p* = 0.04
Tempeh is suitable as a substitute for meat	6.4 (0.2)	6.2 (0.4)	6.3 (0.7)	*p* = 0.92
Was the dish easy to cook?	7.4 (0.2)	7.3 (0.3)	7.0 (0.5)	*p* = 0.15
Was tempeh easy to cook?	7.6 (0.1)	7.9 (0.2)	7.2 (0.4)	*p* = 0.36
Did the dish meet your expectations?	7.4 ^a^ (0.2)	6.7 ^a,b^ (0.3)	5.8 ^b^ (0.5)	*p* = 0.01
Was the appearance appetising?	7.4 (0.2)	6.7 (0.3)	6.8 (0.6)	*p* = 0.08
Was the odour appetising?	6.7 (0.2)	5.8 (0.3)	6.6 (0.6)	*p* = 0.08
What was the balance in flavour and taste like?	7.2 ^a^ (0.2)	5.2 ^b^ (0.3)	5.2 ^b^ (0.6)	*p* < 0.001
Did you find umami in the dish?	6.3 (0.2)	5.6 (0.4)	5.1 (0.6)	*p* = 0.06
What was the balance of the texture like?	7.0 (0.2)	6.2 (0.3)	5.9 (0.5)	*p* = 0.03 *^,1^
How rich did you find the dish in flavour?	7.2 ^a^ (0.2)	6.3 ^b^ (0.3)	5.7 ^b^ (0.6)	*p* = 0.004
Would you try tempeh in other dishes?	6.1 (0.3)	6.1 (0.3)	4.5 (0.7)	*p* = 0.14

* No significant difference was found in a pairwise comparison using the Tukey method for comparison.

**Table 5:** Assessment of a dish with three different types of tempeh rated on a scale from 1 (not at all) to 9 (very much). The number of consumers is given under each dish. The average is given +/− stderr. Different letters in the same line show a significant difference.

	Pizza (*n* = 41)				Filo Dough (*n* = 75)			
	Fava Bean	Pea	Barley	P(dif)	Fava Bean	Pea	Barley	P(dif)
Liking of the dish	4.9 (0.3)	5.0 (0.3)	5.7 (0.3)	0.06	4.8 (0.2)	5.2 (0.2)	5.2 (0.2)	0.22
Liking of tempeh in the dish	4.7 (0.4)	4.6 (0.3)	5.3 (0.3)	0.15	4.3 (0.3)	4.7 (0.3)	4.9 (0.3)	0.12
Tempeh was suitable in the dish	4.4 (0.4)	4.4 (0.4)	4.9 (0.4)	0.21	4.5 (0.3)	4.8 (0.3)	4.9 (0.3)	0.34
Did the dish meet your expectations?	5.8 (0.4)	5.7 (0.4)	6.3 (0.4)	0.14	5.0 (0.3)	5.3 (0.3)	5.2 (0.3)	0.14
Was the appearance appetising?	6.8 (0.3)	7.1 (0.3)	6.8 (0.3)	0.54	6.1 (0.2)	5.9 (0.2)	6.2 (0.2)	0.27
Was the odour appetising?	6.4 (0.3)	6.9 (0.3)	6.4 (0.3)	0.06	6.0 (0.2)	6.4 (0.2)	6.0 (0.2)	0.10
What was the balance in flavour and taste like?	6.0 ^a^ (0.3)	5.0 ^b^ (0.3)	6.4 ^a^ (0.3)	<0.001	4.7 (0.2)	4.6 (0.2)	4.8 (0.2)	0.73
Did you find umami in the dish?	5.4 ^a^ (0.3)	4.7 ^b^ (0.3)	5.7 ^a^ (0.3)	0.01	4.7 (0.3)	4.7 (0.3)	5.0 (0.3)	0.42
What was the balance of the texture like?	6.0 (0.3)	6.3 (0.3)	5.9 (0.3)	0.68	5.2 (0.2)	5.1 (0.2)	5.5 (0.2)	0.31
How rich did you find the dish in flavour?	6.1 (0.3)	6.3 (0.3)	6.5 (0.3)	0.23	4.7 (0.3)	4.7 (0.3)	5.0 (0.3)	0.32
Would you try tempeh in other dishes?	3.7 (0.4)	4.0 (0.4)	4.4 (0.4)	0.26	4.0 (0.3)	4.3 (0.3)	4.5 (0.3)	0.30

## Data Availability

The data presented in this study are available on request from the corresponding author. Although consumer data have been anonymised, data are not publicly available.

## Supplementary Materials

The following are available online at https://www.mdpi.com/article/10.3390/foods10112865/s1, supplementary file: The full questionnaires.
