# Supplementary material for: Introducing Tempeh as a New Plant-Based Protein Food Item on the Danish Market
Source: Foods. 2021 Nov 19;10(11):2865. doi: 10.3390/foods10112865 (PMC8619156; doi:10.3390/foods10112865)
Supplement: Supplementary file 1 [file foods-10-02865-s001.zip › HUT1_1 pers_Marinated fried tempeh.pdf]

Stegt tempeh med grønne asparges, appelsin-miso-sauce, sort sesam og blomkålsris

(1 personer)

Ingredienser

- 100 g tempeh
- 6 stk. (ca. 150 g) grønne asparges
- Olie til stegning

Miso-sauce

- 1 spsk. (ca. 15 g) miso pasta
- 2 spsk. (ca. 20 ml) balsamico eddike
- 2 spsk. (ca. 20 ml) olie
- Saften af ½ stk. appelsin

Blomkålsris

- ¼ stk. (ca. 175 g.) blomkål
- Bunden fra de grønne asparges
- Salt

Pynt/drys

- 1 spsk. sort sesamfrø

Skær tempehen ud i firkanter (ca. 1,5 x 1,5 cm). Skyl de grønne asparges. Skær ca. 3-4 cm af bunden – denne del er 'træet' (smid dem ikke væk, men gem til senere). Del aspargesstænglerne i 3 lige lange stykker.

Steg tempeh ternene på en varm pande med lidt olie til de er gyldne på alle flader – tag af panden. Kom lidt olie på panden igen, og steg de grønne asparges på samme pande, drys med lidt salt. Aspargesene skal stadig have bid, så de skal kun have et par minutter på panden.

Sæt en gryde over med vand og lidt salt. Kog op under låg. Skyl blomkålen og del den i store buketter. Riv blomkålen på den grove side af rivejernet. Kom op i en skål. Nu skal bunden af asparges bruges. Skær dem i tynde skiver, og kom dem op til blomkålen og vend rundt. Når vandet koger, hældes det over blomkåls-aspargesblandingen. Lad trække ca. 1 min. og si vandet fra – tilsæt ¼ tsk. salt og vend rundt.

Bland miso, eddike, olie og appelsinsaft i en skål. Vend de stegte tempeh tern og asparges i miso-appelsinsaucen.

Server tempeh og asparges med blomkålsrisene, hæld evt. resterende miso-appelsinsauce over og drys med sort sesamfrø.

Cut the tempeh into squares (approx. 1.5 x 1.5 cm). Rinse the green asparagus. Cut approx. 3-4 cm from the bottom - this part is the 'tree' (do not throw them away, but save for later). Divide the asparagus stalks into 3 equal pieces.

Fry the tempeh cubes on a hot pan with a little oil until they are golden on all surfaces - remove from the pan. Put a little oil on the pan again, and fry the green asparagus on the same pan, sprinkle with a little salt. The asparagus should still have bite, so they should only have a few minutes on the pan.

Put on a saucepan with water and a little salt. Bring to the boil under a lid. Rinse the cauliflower and divide it into large bouquets. Grate the cauliflower on the coarse side of the grater. Put it up in a bowl. Now the bottom of the asparagus should be used. Cut them into thin slices, and bring them up to the cauliflower and mix around. When the water boils, pour it over the cauliflower-asparagus mixture. Let draw approx. 1 min. and strain the water off - add ¼ tsp. salt and turn around.

Mix miso, balmasic vinegar, oil and orange juice in a bowl. Mix the fried tempeh cubes and asparagus into the miso-orange sauce.

Serve tempeh and asparagus with the cauliflower rice, pour, if necessary, the remaining miso-orange sauce over and sprinkle with black sesame seeds.

!

QR kode til spørgeskema EFTER, du/I har spist retten

Husstandskode
