# Supplementary material for: Introducing Tempeh as a New Plant-Based Protein Food Item on the Danish Market
Source: Foods. 2021 Nov 19;10(11):2865. doi: 10.3390/foods10112865 (PMC8619156; doi:10.3390/foods10112865)
Supplement: Supplementary file 1 [file foods-10-02865-s001.zip › HUT1_1 pers_Nordic inspired summer rolls.pdf]

Nordiske sommerruller med teriyakimarineret tempeh og sursød- og peanutdip.

(1 person)

Ingredienser:

Marinade:
- ½ dl Teriyaki
- ½ fed hakket hvidløg
- ¼ stk. hakket chili
- ¼ spsk. revet ingefær

Fyld:
- 100 g tempeh
- 4 stk. rispapir
- 75 g gulerod (1 stk. mellem størrelse)
- 2 stk. asparges
- 1 forårsløg
- 1/3 stk. agurk
- 50 g rød (spids)kål
- 30 g radiser
- ¼ bdt. koriander
- 1 stk. lime (Til servering)
- Olie til stegning

Peanutbutter dip:
- ½ dl Crunchy peanutbutter
- ¼ dl vand
- ½ stk. lime
- 1 spsk. soya
- salt og peber

Sur-sød dip:
- ½ dl vand
- 2 spsk. sukker
- 2 tsk. eddike
- ¼ stk. chili i skiver
- 2 spsk. fish sauce
- ½ stk. lime

Fremgangsmåde:
Start med at forberede marinaden til tempehen. Bland alle ingredienserne til marinaden sammen, hvorefter tempehen lægges i marinaden.

Imens tempehen suger marinaden til sig, klargøres de øvrige ingredienser til fyldet. Snit gulerødder, asparges, forårsløg, agurk, rød spidskål og radiser i strimler og læg dem klar på et skærebræt eller fad.

Peanutbutter dip: Bland alle ingredienserne sammen og sæt til side til servering.

Sur-sød dip: Bland alle ingredienser sammen og sæt til side til servering.

Hæld lidt olie på en stegepande.

Tag nu tempehen op af marinaden, og skær den ud i strimler af omkring 0,5x1 centimeters tykkelse. Steg dem et par minutter på panden til de bliver gyldne. (OBS. Gem evt. resten af marinaden til dip)

Opblød rispapir i koldt vand (det tager ca. 1 min.), og læg det herefter over på en tallerken, hvor sommerrullerne samles. Læg et par stykker af hver strimmel grøntsag samt omkring 2 strimler tempeh i hver sommerrulle. Læg korianderblade i og rul den stramt sammen. Skær et par limebåde ud til at presse over sommerrullerne, når de skal spises. Server med de 2 typer dip.

!

QR kode til spørgeskema <u>EFTER</u>, du/I har spist retten

Husstandskode

Directions:

Start by preparing the marinade for the tempeh. Mix all the ingredients for the marinade together, after which the tempeh is added to the marinade.

While the tempeh is absorbing the marinade, the other ingredients are prepared for the stuffing. Cut carrots, asparagus, spring onions, cucumber, red cabbage and radishes into strips and place them ready on a cutting board or platter.

Peanutbutter dip: Mix all the ingredients together and set aside for serving.

Sour-sweet dip: Mix all ingredients together and set aside for serving.

Pour a little oil on a frying pan.

Now take the tempeh out of the marinade and cut it into strips about 0.5x1 centimeters thick. Fry them for a few minutes on the pan until golden. (NOTE. If necessary, save the rest of the marinade for dip)

Soak rice paper in cold water (it takes about 1 min.) and then place it on a plate where the summer rolls will be assembled. Put a few pieces of each strip of vegetable as well as about two strips of tempeh in each summer roll. Put cilantro leaves in and roll it tightly. Cut out a couple of lime boats to squeeze over the summer rolls when they are to be eaten. Serve with the 2 types of dip.
