# Supplementary material for: Introducing Tempeh as a New Plant-Based Protein Food Item on the Danish Market
Source: Foods. 2021 Nov 19;10(11):2865. doi: 10.3390/foods10112865 (PMC8619156; doi:10.3390/foods10112865)
Supplement: Supplementary file 1 [file foods-10-02865-s001.zip › HUT1_1 pers_Tempeh-'meat balls'.pdf]

Tempeh-deller med persille og hasselnødder serveret i tomatsauce med pasta

(1 person)

<u>Ingredienser</u>

Tempeh-deller

- 100 g tempeh
- ¼ bdt. bredbladet persille
- ¼ fed hvidløg
- ¼ stk. løg
- 10 g hasselnødder
- Ca. 2 spsk. vand
- En knivspids salt
- ½ spsk. olie (til at smøre på bageplade/bagepapir)

Tomat sauce

- 1 ds. flåede tomater
- ¾ stk. løg
- 1 ¾ fed hvidløg
- 3 stk. persillestilke (fra tempeh-dellerne)
- ½ spsk. olie
- ½ tsk. salt

- 100 g fuldkornspasta
- ½ tsk. salt

Indstil ovnen til 185 grader (varmluft).

Skyl persillen grundigt. Pluk persillen groft fra stilken (gem 3 stilke til tomatsaucen). 1/3 hakkes fint, og gemmes til servering. Resten bruges i tempeh-dellerne.

Hvis du/I har en blender/minihakker, så blend alle ingredienser til tempeh-dellerne i den - på nær olien; den skal bruges til at smøre en bageplade eller bagepapir med. Har du/I ikke en blender/minihakker, så hak alle ingredienser meget fint (også tempehen). Bland det hele i en skål og tilsæt vand – bland/mos sammen med en gaffel (teksturen bliver lidt grovere på denne måde).
Tempeh-mosen formes til små kugler (ca. 2,5 x 2,5 cm) – for at undgå, at mosen klistrer fast i hånden kan man ruller, kan smøre dem med lidt vand eller olie – så slipper de lettere hånd fladen. Sæt tempeh-kuglerne på en smurt ovnplade eller et stykke smurt bagepapir, og sæt pladen ind midt i ovnen. De skal have ca. 20 min. i alt, men vend dem gerne et par gange.

Hak løg og hvidløg og steg dem gyldne i olien i en gryde. Tilsæt de flåede tomater, persillekviste og salt. Lad saucen simre i ca. 15 min. Inden servering tages persillestilkene op.

Sæt en gryde med vand og salt over med låg og kog op (til pastaen). Kog pastaen til den er mør, men stadig har bid. Sigt vandet fra.

Server pastaen med tomatsauce og tempeh-deller; slut af med et drys hakket persille.

Set the oven to 185 degrees (convection).

Rinse the parsley thoroughly. Roughly, pick the parsley from the stem (save three stalks for the tomato sauce). Finely chop 1/3 of it, and save it for serving. The rest will be used in the tempeh 'meatballs'.

If you have a blender / mini chopper, blend all the ingredients for the tempeh 'meatballs' in it - except the oil; it should be used to grease a baking tray or baking paper. If you do not have a blender / mini chopper, then chop all the ingredients very finely (also the tempeh). Mix it all in a bowl and add water - mix / mash together with a fork (the texture gets a little bristlier this way).

The Tempeh-mix is shaped into small balls (approx. 2.5 x 2.5 cm) - to prevent the mix from sticking to the hand, you can roll, grease them with a little water or oil - then they release the hand surface more easily. Place the tempeh balls on a greased baking sheet or a piece of greased baking paper, and insert the tray in the middle of the oven. They should bake approx. 20 min. in total, but feel free to flip them a few times.

Chop onions and garlic and fry them golden in oil in a pan. Add the peeled tomatoes, parsley stalks and salt. Let the sauce simmer for approx. 15 min. Before serving, pick up the parsley stalks.

Put a pot of water and salt over with a lid and bring to the boil (for the pasta). Cook the pasta until it is tender but still has bite. Sift the water.

Serve the pasta with tomato sauce and tempeh parts; finish with a sprinkle of chopped parsley.

!

QR kode til spørgeskema EFTER, du/I har spist retten

Husstandskode
