# Supplementary material for: Introducing Tempeh as a New Plant-Based Protein Food Item on the Danish Market
Source: Foods. 2021 Nov 19;10(11):2865. doi: 10.3390/foods10112865 (PMC8619156; doi:10.3390/foods10112865)
Supplement: Supplementary file 1 [file foods-10-02865-s001.zip › HUT1_Survey.pdf]

HUT 1 – Survey: Tempeh

Pre-test: Recruting schedule

Schedule structure	Scale/organization
Samtykke til opbevaring af personfølsomme oplysninger (In Danish)<br>Consent to collecting and keeping of personal information	Yes/no
Info text:<br>You will receive a meal box that contains ingredients and a recipe for a dish with tempeh (evening meal). The dish is vegan.<br>In the meal box, there will be ingredients to prepare a meal for the number of people in the household (1, 2, 3 or 4 people). It will not be possible to participate with children, as the dish must be assessed in a questionnaire afterwards.<br>You choose on the following pages which dish you want and number.<br><br>Prerequisite for participation:<br>• You must be between 18 and 29 years old<br>• You can order for 1, 2, 3 or 4 people in the same household<br>• Everyone in the household must fill in a questionnaire after eating the dish<br>• A photo of the finished dish must be uploaded to Instagram (if you have an account) with #tempehforsøgabsalon<br>If you have the following allergies or intolerances, you cannot participate: celiac disease and / or gluten intolerance, wheat and other grains, soy, nuts, peanuts, fish and shellfish / molluscs, cross-allergic reactions to pollen and latex.<br><br>I want to sign up my household to try a dish with tempeh	Yes/no
Choose how many people between the ages of 18 and 29 who will participate in the household	1, 2, 3, 4
Choose the dish you want to cook	1) Nordic summer rolls with teriyaki-marinated tempeh and sweet and sour peanut dip.<br>2) Fried tempeh with green asparagus, orange miso-sauce, black sesame and cauliflower rice.<br>3) Tempeh 'meatballs' with parsley and hazelnuts served in tomato sauce with pasta.
Choose the reason for your dish of choice (you can choose several)	CATA<br>- It sounds good<br>- It sounds exciting<br>- It sounds like something I/we know<br>- I/we are curious<br>- I/we think that this is the one I/we would like best

	- It sounds easy to make<br>- It sounds healthy<br>- This is something I/we have seen/heard about in the media<br>- I/we have cooked it before<br>- I/we like the sides<br>- Write yourself ________
Choose the reasons why you did not choose one of the other dishes (you can choose several)	- They do not sound good<br>- They sound boring<br>- They sound like something I/we do not know<br>- I do not think I/we like them<br>- They sound difficult to make<br>- They sound unhealthy<br>- I/we have not made them before<br>- I/we have made them before<br>- I/we do not like the sides<br>- Write yourself ________
Pick the day you would like to try a dish with tempeh<br>The meal box will be delivered between 14-17 o'clock on the chosen day and must be cooked the same day.	Monday d. 25/5 2020 – (ONLY Region Sjælland)<br>Tuesday d. 26/6 2020 (Region sjælland AND Region Hovedstaden)<br>Wednesday d. 27/5 2020 (ONLY Region Hovedstaden)
Enter your name and delivery address	
If you have special instructions in connection with delivery, state them here (eg entrance is in the yard, etc.)	
Enter your e-mail address<br>It will be used for sending out a confirmation that you are involved in the trial and on the selected delivery date.	

Post-test: Survey

**Questionaire**	**Scale**
**Consent**	Yes/no
**Personal information**<br>*What is your gender?*	 Woman, man, enter youself____, do not want to submit an answer
Age	18 - 21<br>22 - 25

	26 - 29
Place of residence	Region Sjælland<br>Region Hovedstaden
Education	Primary school or equivalent<br>Apprenticeship or apprenticeship in a subject<br>High school education<br>Short higher education (under 3 years)<br>Medium-term higher education (3-4 years)<br>Long higher education (over 4 years)
Did you participate in the preparation of the dish with tempeh?	Yes/No
**Consumer evaluation of general liking of the exemplary dish**<br>*To what extent did you like the dish as a whole?*	Hedonic<br>9 pkt.
**Consumer evaluation of general liking of tempeh in an exemplary dish**<br>*To what extent did you like tempeh in the dish?*	Hedonic<br>9 pkt.
**Consumer evaluation of suitability in the dish**<br>*To what extent do you think tempeh fits into the dish?*	Hedonic<br>9 pkt.
**Meat substitute**<br>*To what extent do you think tempeh is a good substitute for animal protein sources?*	Hedonic<br>9 pkt.
**Preparation of the dish**<br>*To what extent do you think the dish was easy to prepare?*	Hedonic<br>9 pkt. + det var ikke mig, der lavede maden
**Preparation of tempeh**<br>*To what extent do you think the tempeh was easy to cook?*	Hedonic<br>9 pkt. + det var ikke mig, der lavede maden
**Consumer evaluation of the culinary success factors of the dish**<br>*- To what extent did the name and description of the dish live up to your expectations?*<br>*- To what extent do you think the dish had an appetizing appearance?*<br>*- To what extent do you think the dish had an appetizing smell?*<br>*- To what extent do you think the dish had a good balance between sweet, salty, sour, bitter and umami (umami is what gives an experience of e.g. power, roundness, depth, strong broth)*<br>*- To what extent do you think there was umami present in the dish (umami gives a taste and experience of e.g. power, roundness, depth, strong broth)?*<br>*- To what extent do you think there was a good balance between the different textures in the dish?*<br>*- To what extent do you think the dish was rich in flavor?*	Hedonic<br>9 pkt.
**Consumer evaluation of trying tempeh in a dish again**<br>*To what extent would you try to use tempeh again in other dishes?*	Hedonic<br>9 pkt.
**Prior knowledge of tempeh**<br>*Did you know about tempeh prior to this study?*	Binomial<br>Yes/no
**Prior use of tempeh**<br>*Have you used tempeh in cooking before this study?*	Binomial<br>Yes/no

- *If yes: Where do you buy your tempeh?*<br>- *If no: Why not?*	 -
**Prior use of other vegetarian protein sources**<br>*In the past week, have you used vegetarian protein sources other than tempeh in the preparation of dinner?*<br>- *If yes: enter the 2 most commonly used*	Binomial<br>Yes/No<br><br>-
**Elaboration / possibly**<br>*Is there anything you would like us to know about tempeh and the dishes that we have not asked?*	
**Ending**<br>'Thank you so much for the help'<br>The project is funded under Data- and Design-Driven Health Innovation (DDSI).<br>The Tempeh comes from Contempehrary, which is a local producer of tempeh made from Nordic ingredients.<br>You can read more about Contempehrary here:<br>https://www.contempehrary.com/	
