# Supplementary material for: Introducing Tempeh as a New Plant-Based Protein Food Item on the Danish Market
Source: Foods. 2021 Nov 19;10(11):2865. doi: 10.3390/foods10112865 (PMC8619156; doi:10.3390/foods10112865)
Supplement: Supplementary file 1 [file foods-10-02865-s001.zip › HUT2_1 pers_Pizza.pdf]

Pizza med tempeh, kartoffel, jordskokker og syltede rødløg toppet med sprød marineret grønkål

(1 person)

Ingredienser:

Pizzadej:
- 150 g hvedemel tipo 00
- 5 g gær
- 1 dl vand
- 1 spsk. olivenolie
- ½ tsk. salt

Pizzafyld:
- 30 g (3 skiver) ærtetempeh
- 30 g (3 skiver) bønnetempeh
- 30 g (3 skiver) korntempeh
- 2 spsk. olivenolie
- 1 stk. (ca. 220 g) bagekartoffel
- 125 g jordskokker
- 1 fed hvidløg
- 4 stilke bredbladet persille
- ½ dl olivenolie

Syltede rødløg
- 1 – 2 stk. rødløg (150 g)
- 1 spsk. olie
- 40 g sukker
- ½ dl lagereddike
- Salt, peber

Sprød marineret grønkål:
- 40 g grønkål (uden stok)
- 1 spsk. olivenolie
- ½ spsk. eddike
- ½ tsk. sukker
- ¼ tsk. sennep (grov)
- Salt, peber

Fremgangsmåde:
Pizzadej: Udblød gæren i lunken vand. Tilsæt olivenolie og salt. Herefter tilsættes melet. Ælt dejen godt igennem. Sæt dejen til hævning i 1 ½ time.

Kartoffelfyld: Skyl kartoflerne (skal ikke skrælles) og skær dem i tynde skiver. Skyl jordskokkerne grundigt (skræl dem kun, hvis de er meget beskidte med jord), og skær dem i tynde skiver. Skyl persillen og pil hvidløgene. Hak persille og hvidløg og bland med olivenolie og lidt salt og peber.

Kog kartoffel- og jordskokkeskiverne i ca. 3 min. i letsaltet vand. Køl dem hurtigt ned under rindende koldt vand. Hæld vandet fra og lad dem dryppe af. Vend kartoffel-jordskokkeblandingen med persille-hvidløgsolien. Smag til med salt og friskkværnet peber.

Syltede rødløg: Pil løgene, og skær dem i skiver. Sauter dem ved svag varme i olivenolie, til de er faldet sammen. Hæld eddike på og tilsæt sukker, salt og peber. Skru ned for varmen og lad løgene simre, til de smelter sammen til en mørk-lilla løgmarmelade.

Tempeh: Skær de 3 typer af tempeh ud i skiver (ca. ½ cm i tykkelsen). Vend skiverne i olivenolie (se vejledning med billeder)

Samling af pizza: Rul dejen tyndt ud til 1 pizzabund på et meldrysset bord. Dejen lægges over på bagepapir på ovnristen. Hvis der går hul i dejen, så bare lap den med lidt dej fra kanten. Det betyder ikke noget.

Fordel kartoffel-jordskokke- og persilleblandingen i et tyndt lag på pizzabunden. Bag pizzaen ved 220 grader i ca. 10 min. Tag pizzaen ud og fordel herefter tempehskiverne på pizzaen – (se vejledning med billeder). Afslut med at fordele de syltede rødløg på toppen – drys med groft salt. Bag pizzaen ved 220 grader i yderligere ca. 10 min.

Sprød marineret grønkål: I mens pizzaen bager, skylles grønkålen og den grove stilk skæres fra. Hak grønkålen fint. Bland olie, eddike, sukker, sennep, salt og peber sammen i en skål. Ved det sammen med grønkålen.

Når pizzaen er taget ud af ovnen dryssen den sprøde marinerede grønkål hen over.

HUSK at notere hvilken tempehtype du/I spiser først, næst og sidst, så du/I kan huske det, når spørgeskemaet skal udfyldes (se vejledning).

!

QR kode til spørgeskema <u>EFTER</u>, du/I har spist retten

Husstandskode

Directions:

Pizza dough: Soak the yeast in lukewarm water. Add olive oil and salt. Then add the flour. Knead the dough well. Leave the dough to rise for 1 hour.

Potato stuffing: Rinse the potatoes (do not peel) and cut them into thin slices. Rinse the Jerusalem artichokes thoroughly (peel them only if they are very dirty with soil) and cut them into thin slices. Rinse the parsley and peel the garlic. Chop parsley and garlic and mix with olive oil and a little salt and pepper.

Boil the potato and Jerusalem artichoke slices for approx. 3 min. in lightly salted water. Cool them down quickly under running cold water. Pour off the water and let them drip off. Mix the potato-artichoke mixture with the parsley-garlic oil. Season with salt and freshly ground pepper.

Pickled red onions: Peel the onions and cut them into slices. Sauté over low heat in olive oil until they have collapsed. Pour on vinegar and add sugar, salt and pepper. Turn down the heat and let the onions simmer until they melt together into a dark-purple onion jam.

Tempeh: Cut the three types of tempeh into slices (approx. ½ cm in thickness). Mix the slices with olive oil (see instructions with pictures)

Collection of pizza: Roll out the dough thinly to 1 pizza base on a floured table. Place the dough on baking paper on the oven rack. If there is a hole in the dough, just pat it with a little dough from the edge. It does not mean anything.

Spread the potato-artichoke and parsley mixture in a thin layer on the pizza base. Bake the pizza at 220 degrees for approx. 10 min. Take out the pizza and then distribute the tempeh slices on the pizza - (see instructions with pictures). Finish by spreading the pickled red onions on top - sprinkle with coarse salt. Bake the pizza at 220 degrees for a further approx. 10 min.

Crispy marinated kale: While the pizza is baking, rinse the kale and cut off the coarse stalk. Finely chop the kale. Mix oil, vinegar, sugar, mustard, salt and pepper together in a bowl. Know it along with the kale.

Once the pizza is taken out of the oven, sprinkle the crispy marinated kale over it.

REMEMBER to note which type of tempeh you eat first, second and last, so that you can remember it when filling out the questionnaire (see instructions)
