# Supplementary material for: Introducing Tempeh as a New Plant-Based Protein Food Item on the Danish Market
Source: Foods. 2021 Nov 19;10(11):2865. doi: 10.3390/foods10112865 (PMC8619156; doi:10.3390/foods10112865)
Supplement: Supplementary file 1 [file foods-10-02865-s001.zip › HUT2_1_pers_Tempeh in filo dough.pdf]

Tempeh in filo dough

Directions:

Potato stuffing: Rinse the potato (do not peel) and grate it on the rough side of a grater. Peel the pumpkin, grate it and squeeze the juice. Cut it into cubes of ½ x ½ cm. Peel and slice the onion. Cut the mushrooms into slices. Rinse and chop the parsley coarsely (also the stem). Heat a pan with oil. Fry all the ingredients - except the parsley over medium heat - flip often. When the pumpkin is almost tender, add parsley, salt and pepper. Mix around and take it off the pan.

Tempeh: Now put oil on the pan again (it is not necessary to wash off the pan) and heat the pan. Cut the tempeh into slices (approx. 1 cm thick). Fry the tempeh slices approx. 1 min. on each side (see instructions with pictures).

Filo dough: Preheat the oven to 200 degrees. Divide the filo dough so that there are 3 sheets. Per person. On a piece of baking paper, lay the first sheet of filo dough with the narrow side down towards you. Brush the entire sieve with oil. Now turn the page over so that the oil side is facing down against the baking paper. Place approx. 3 tbsp. of stuffing at the beginning of the filo dough sheet. Place 2 slices of tempeh on top and roll the filo dough with the stuffing like a spring roll. Finish by bending the ends under the roll (see instructions with pictures regarding keeping track of tempeh type). Repeat for all sheets. Bake at 200 degrees for approx. 15-20 min.

Sauce vert (green dressing): While the filo dough rolls are in the oven, rinse and chop the parsley and mint finely. Mix oil, sugar, salt and pepper with vegan yogurt. Add the chopped herbs.

Serve the filo dough tempeh roll with sauce vert.

REMEMBER to note which type of tempeh you eat first, second and last, so that you will remember it when the questionnaire is to be answered (see the instructions).

!

QR kode til spørgeskema EFTER, du/I har spist retten

Husstandskode
